# The Impact of Socioeconomic Determinants on the Quality of Life of Moroccan Breast Cancer Survivors Diagnosed Two Years Earlier at the National Institute of Oncology in Rabat

**DOI:** 10.1155/2021/9920007

**Published:** 2021-06-23

**Authors:** Rachid Ismaili, Leila Loukili, Hind Mimouni, Imane EL Haouachim, Abderraouf Hilali, Bouchra Haddou Rahou, Rachid Bekkali, Ahmed Nejmeddine

**Affiliations:** ^1^Hassan First University, Settat, Morocco; ^2^Fondation Lalla Salma Prevention and Treatment of Cancers, Rabat, Morocco; ^3^Research Department, High Institute of Nursing Professions and Technical Health, Rabat, Morocco

## Abstract

**Introduction:**

The objective of this study was to investigate the impact of socioeconomic determinants on the quality of life of Moroccan women with breast cancer two years after their diagnosis who are followed up at the National Institute of Oncology (INO) in Rabat.

**Methods:**

This is a cross-sectional study that was conducted between May 2019 and September 2020. The sample size was 304 women. Data were collected using the EORTC QLQ-C30 and EORTC QLQ-BR 23 questionnaires in the Moroccan dialect.

**Results:**

The mean age of participants was 53.5 ± 12.4 years, where the majority resided in urban areas and more than half were illiterate. Moreover, three-quarters of the survivors were not working, and almost all have basic medical coverage. Nearly one-third of the respondents had experienced discrimination from those around them, and nearly half attributed the decrease in income to their state of health. In addition, 38.2 percent of participants stated that they had great difficulty living on their monthly income after the illness, whereas more than half of the survivors had a good quality of life in terms of overall health (GHS/QOL). Besides, social function obtained the highest score, while emotional function obtained the lowest score. Furthermore, financial difficulty was the most distressing symptom. Indeed, income adjustment after the disease, discrimination, distance between home and treatment center, professional status, and medical coverage were correlated with GHS/QOL. Regression analysis revealed that income adjustment after illness and discrimination were significant predictors of GHS/QOL.

**Conclusion:**

The data suggest establishing a financial support program and the development of education and awareness-raising policies to combat discrimination.

## 1. Introduction

The cancer incidence rate in Morocco is 139.6 cases per 100,000, and the mortality rate is estimated to be 86.9 per 100,000. In addition, Morocco has registered 59,370 new cancer cases, of which breast cancer constitutes 11,747 cases or 19.8% [1]. Survival of breast cancer patients has improved significantly due to early detection and advances in oncology treatment [2, 3]. The average 5-year survival rate has been estimated to be 85% in developed countries, but only 50–60% in developing countries [4, 5]. In Morocco, according to the Rabat Cancer Registry at the National Institute of Oncology, the overall survival was 97.1% at one year, 89.2% at three years, and 80.6% at five years [6]. On the other hand, according to data published in the latest Greater Casablanca Cancer Registry Report 2008–2012, the overall survival at 5 and 7 years for breast cancer cases was 79% and 65%, respectively [7]. While there are advances in diagnosis and treatment, it still has adverse effects on social and physical functioning [4, 5]. In other words, breast cancer diagnosis and treatment affect quality of life, physical functioning, and psychological well-being [2]. Indeed, a mastectomy has a wide range of functional and emotional consequences, such as depression, which can be as prevalent as 56% in Western countries [4], as well as axillary curage, which increased the risk of disability benefits or loss of paid employment during the first five years of follow-up [8].

Cancer management requires extensive and expensive treatment that deteriorates the quality of life of survivors [9]. A US study showed that the direct costs associated with breast cancer, between 3 and 24 months, were US$131 per month. These expenses cover catering, transportation, telephone calls, housekeeping and laundry services, childcare, and hotel stay. These costs represented 6% of income for women earning less than US$30,000 per year and only 2% for those earning US$60,000 per year [10], and the low income was associated with poor quality of life [11, 12]. For example, financial hardship is considered a significant adverse effect of cancer treatment and is associated with reduced quality of life [13, 14]; indeed, 16% to 78% of cancer survivors experienced treatment-related financial hardship [15, 16]. Despite 100% coverage, hidden costs persist, with 47% of cancer survivors who have been treated for cancer reporting that they have had out-of-pocket health care costs, 8% of which were significant [17]. In addition, several research studies suggest that the diagnosis and treatment of breast cancer have adverse effects on the physical, psychological, and social health of patients and may reduce their quality of life [18–20]. Indeed, patients receive mastectomy or breast conservative treatment; they feel stigmatized [19]. Furthermore, disease-related stigma can take many different forms, including discriminatory behavior from others [21, 22]. In other words, 12.0% of employees report having experienced rejection or discrimination directly related to their cancer from coworkers [23].

The literature review suggested many studies that have addressed the quality of life of women with long-term breast cancer, 5 to 10 years, after diagnosis and treatment, but few have studied the impact of diagnosis and treatment between the first and fourth year after cancer on the study of the quality of life [24, 25]. The present study is part of this perspective and strives to study the impact of socioeconomic determinants on the quality of life of Moroccan breast cancer survivors, who are followed up at INO Rabat, two years after their diagnosis. The results obtained constitute a database on breast cancer and may expose discoveries that will eventually help other authors in different breast cancer research.

## 2. Materials and Methods

### 2.1. Study Design

This is a cross-sectional study aimed at examining the impact of socioeconomic determinants and the quality of life of Moroccan breast cancer survivors, who are being followed up at INO in Rabat, two years after their diagnosis. Three hundred four (304) breast cancer survivors were selected during their follow-up consultation at INO Rabat between May 1 and September 30, 2020. This study is part of a thesis entitled “The Social Cost of Cancer: Impact of Breast and Lung Cancer on the Quality of Life of the Patient and Her Nuclear Family after Two Years of Diagnosis.”

### 2.2. Inclusion Criteria

The study included all survivors of breast cancer diagnosed two years earlier who are being followed up at INO Rabat, married with children, and at all stages. They did not present any physical or mental illness.

### 2.3. Exclusion Criteria

The study excluded all survivors who are single and survivors with a history of physical or mental disorders or other types of pathology other than breast cancer.

### 2.4. Data Collection

The designated nurse interviewer identified eligible study participants at the recruitment site (Consultation Units). The nurse checked the inclusion criteria, answered survivors' questions, and resolicited their consent to participate. Once consent was obtained from participants, clear information about the study and its objectives was provided.

### 2.5. Ethical Considerations

Approval to conduct this study was obtained from the Ethics Committee for Biomedical Research Mohammed V University (Rabat Faculty of Medicine and Pharmacy, Rabat Faculty of Dental Medicine) (N/R: Folder Number 63/19).

### 2.6. Instruments

This work used the EORTC QLQ-30 Quality of Life Questionnaire as a data collection tool, combined with the EORTC QLQ-BR23 add-on module, in order to establish a standardized measure of the various aspects of quality of life.

### 2.7. EORTC Questionnaire QLQ-30

This tool was designed in 1986 by the European Cancer Treatment Research Organization. It has been validated in numerous tumor localizations. It includes thirty (30) items divided into five functional scales (physical, role, cognitive, emotional, and social), three symptomatic scales (fatigue, pain, nausea, and vomiting), and global health and quality of life scale. It has been tested in the United States, Australia, Europe, and Japan and has demonstrated high reliability and validity across continents [26, 27]. The explored dimensions include between one to five different items. The results of these different scales allow the calculation of a score out of 100, which is illustrated in the procedure described in the EORTC QLQ-30 Scoring Manual. A high score for a functional scale reflects an optimal function of the measured variables. On the other hand, a high score for a symptomatic scale reflects a high level of symptoms. In contrast, a high overall health score explains a good state of health and quality of life.

### 2.8. EORTC QLQ-BR23

The EORTC QLQ-BR23 questionnaire is a complementary module, which is specific to breast cancer. It comprises twenty-three (23) items distributed as follows: four functional scales exploring body image, sexual activity, sexual pleasure, outlook and four symptomatic scales exploring therapeutically specific side effects, breast symptoms, brachial symptoms, and concern about hair loss. The results are interpreted in the same way as before, except for the scales concerning sexual activity and sexual pleasure, for which a high score would indicate a low level of symptoms in contrast to the other symptomatic scales [26]. The transcultural adaptation of the two questionnaires was done and validated in Morocco [28, 29].

### 2.9. Statistical Analysis

In order to achieve the objectives of the study, a descriptive analysis of the sociodemographic situation was carried out with the calculation of statistical parameters such as means and standard deviations. A simple linear regression model was applied to detect the association between GHS/QOL and socioeconomic characteristics. Variables with *p* ≤ 0.20 on univariate analysis were included in the regression model to assess predictors of overall health-related quality. All confounding variables were included in the multivariate analysis. The results of the multivariate analysis are presented as *β* with a *p* ≤ 0.05 being considered statistically significant. The scoring of the EORTC QLQ-C30 items was performed according to the EORTC scoring manual [30]. Statistical analysis was performed using the SPSS version software. In case of missing items, multi-items scores were calculated as the mean of nonmissing items if at least half of the items from the corresponding scale had been completed.

## 3. Results

### 3.1. Sociodemographic Characteristics

A total of 304 women were included in the study between May 2019 and September 2020.

The average age was 53.5 ± 12.4 years with extremes of 23 and 85 years. The age range (46–65 years) is the most dominant with 61.8%. More than half of the survivors resided in urban areas (69.7%), and the majority of them live within 50 km of the treatment center (40.8%). Besides, 36.2% live between 51 and 200 km away, while 20.4% live between 201 and 350 km away. More than half of the women were illiterate (52%), 27% had completed primary school, and 75% have no professional activity. In addition, more than half of the women are affiliated with the RAMED (68.4%), whereas the rest are divided, respectively, between CNOPS, CNSS, and insurance (20.4%, 10.2%, and 0.3%). Women who had no monthly income before the disease represent 43.4%, whereas 37.5% had a monthly income below 2500DHS. 24.7% reported having experienced discrimination from their entourage.

Moreover, most survivors reported that their monthly income after the illness was stable (61.8%), while 33.6% confirmed that their monthly income had decreased. 48.7% of participants attributed this decrease to their state of health, while 38.2% of survivors reported great difficulty living on their monthly income after the illness.  Table 1 illustrates these characteristics in detail.

### 3.2. Quality of Life: EORTC QLQ-C30

The survivors' quality of life assessment was done two years after diagnosis, where the various parameters of the EORTC QLQ-C30 and EORTC QLQ-BR23 questionnaires were evaluated. The EORTC QLQ-C30 showed that survivors scored fairly well on GHS-QOL scale (mean = 57.2 ± 25.4). Scores on the functioning scales ranged from 51.2 ± 31.3 for emotional functioning to 84.5 ± 29.3 for social functioning. More than half of the survivors were identified as having financial problems related to the disease and treatment with a mean of 54.1 ± 39.9, whereas symptoms pain (34.3 ± 32.6), fatigue (33.3 ± 30.1), dyspnea (32.0 ± 38.7), and insomnia (27.9 ± 37.1) were less distressing for the survivors. Conversely, the symptoms of nausea and vomiting (11.7 ± 26.4), diarrhea (12.1 ± 25.6), constipation (12.9 ± 28.8), and loss of appetite (19.3 ± 32.6) were not a problem for the majority of survivors (Table 2).

### 3.3. Quality of Life: EORTC QLQ-BR23

For the EORTC QLQ-BR23, it was found that all functional scales had mean scores above 50.0, except sexual functioning, which had a mean score of 49.7 ± 29.7. In terms of symptom scales, mean scores ranged from 22.2 to 45.2. The worst symptom was brachial symptoms (45.2 ± 33.4) followed by breast symptoms (35.7 ± 28.2). In contrast, hair loss symptoms (22.2 ± 36.8) and therapeutically specific side effects (27.9 ± 27.7) had the lowest scores (Table 3).

### 3.4. Multiple Linear Regression between Overall Health Status/QOL and Socioeconomic Characteristics of Breast Cancer Survivors

The results in Table 4 show a strong correlation between several socioeconomic characteristics and GHS/QOL. Correlated variables were adjustment in income after the illness (*p* ≤ 0.001), discrimination (*p* ≤ 0.001), distance from home to a treatment center (*p*=0.015), occupational status (*p*=0.047), medical coverage (*p*=0.050), and changes in monthly income after the illness (*p*=0.106). Multivariate analysis revealed that adjustment of monthly income after illness (*p* ≤ 0.001) and discrimination (*p* ≤ 0.001) were significant predictors of GHS/DVQ (QLQ-C30).

**Figure 1:**
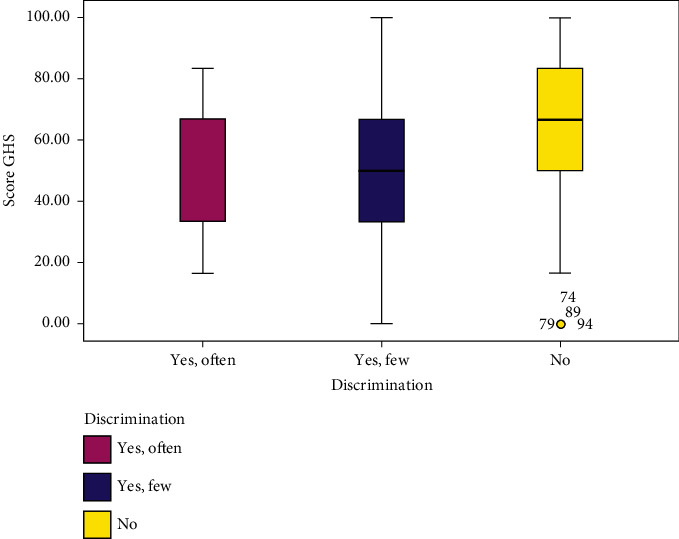
Description of the GHS according to the discrimination status (*p* ≤ 0.001).

**Figure 2:**
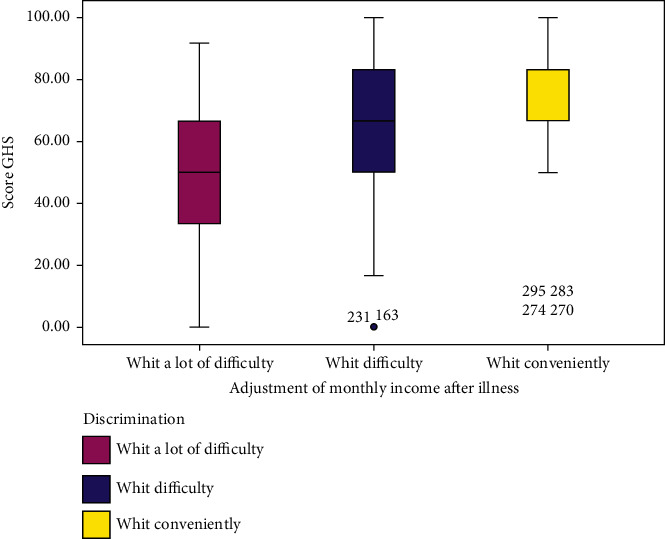
Description of the GHS according to the adjustment of monthly income after illness (*p* ≤ 0.001).

Figure 1 shows that survivors who did not experience discrimination had a higher overall quality of life than those who experienced discrimination often or a few times, respectively (*M*  = 63[*Q*1 = 50, *Q*3 = 82]; *M*  = 50[*Q*1 = 37, *Q*3 = 63]; *M*  = 37[*Q*1 = 37, *Q*3 = 63]; *p* ≤ 0.001). Similarly, Figure 2 shows that survivors who had a monthly income that allowed them to live conveniently had a higher overall quality of life than those who reported a lot of difficulties or difficulty living on their monthly income after illness, respectively (*M* = 83[*Q*1 = 68, *Q*3 = 83]; *M* = 67[*Q*1 = 50, *Q*3  = 83]; *M* = 50[*Q*1 = 33, *Q*3 = 92]; *p* ≤ 0.001).

## 4. Discussion

Indeed, some studies have been carried out at the national level and particularly at INO on the quality of life of women with breast cancer, either during treatment or after one year of diagnosis [31, 32]. However, to our knowledge, the present study is the first initiation conducted in Morocco on the quality of life in women with breast cancer diagnosed two years earlier. All patients were included in the study regardless of their stage of breast cancer. Mierzynska et al. updated the reference values (RV) for the EORTC QLQ-C30 in early and metastatic breast cancer. For early breast cancer, RV EORTC revealed high functioning and low prevalence of symptoms, while RV from metastatic breast cancer had lower baseline Health-Related Quality of Life (HRQoL) values than those from early breast cancer, and cognitive functioning presents the highest mean scores, while role functioning presents the lowest mean score. In addition, in the symptom scales, metastatic breast cancer presents a low prevalence of nausea/vomiting and diarrhea and a high prevalence of fatigue and pain, while HRQoL was more impaired in patients with metastatic breast cancer than in the general healthy population [33].

Our results attest that more than half of the survivors had a mean GHQ (57.2 ± 25.4), which is similar to the mean score of metastatic breast cancer (57.6 ± 23.1) and lower than the mean score of early breast cancer (76.9 ± 19.2) [33]. In addition, the survivors had physical (72.6 ± 28.0), social (84.5 ± 29.3), and emotional (51.2 ± 31.3) functioning that has lower values than the baseline EORTC QLQ-C30 for early breast cancer, respectively ((92.2 ± 12.2); (92.2 ± 15.9); (69.5 ± 24.0)) [33]. In the same perspective, the physical role and emotional functional scales in the study survivors are worse than the functional scales in the Härtl et al. study [34]. Improvement in emotional functioning occurred primarily in the first year of follow-up, with less significant differences for the two and three years [35]. In contrast, social and cognitive functions are approximately similar to a previous study [34]. However, the data on social functioning revealed by Kornblith et al. contradict those of the present study [36].

For the EORTC QLQ-C30 symptom scales, mean scores ranged from 11.7 to 54.1. Survivors suffered from fatigue, pain, dyspnea, and insomnia. These symptoms persisted for many years after surgery [37, 38]. Other symptoms did not present significant problems for the survivors, but they are somewhat elevated compared to other studies [34]. Conversely, more than half of the survivors had a change in financial status (mean score = 54.1 ± 39.9) during the two years of follow-up. This score was higher than the score demonstrated by Arndt et al. in breast cancer survivors diagnosed three years earlier [39].

Analysis of the functional dimensions of the EORTC QLQ-BR23 disclosed that 50.7% of survivors do not have a problem with body image (mean = 58.7 ± 31.7). This score is slightly lower than those of the United Kingdom (mean = 78.1 ± 25.8) [40] and Germany (mean = 73.7 ± 30.6) [39, 41]. Similarly, the scores for future prospects and sexual functioning are better than those revealed by Arndt et al. Conversely, scores for sexual enjoyment and body image are worse [39]. In other words, the scores in this study are worse than those found for Kuwaiti women [42]. The results obtained for brachial and mammary symptoms are more altered than those indicated by the authors [34, 39]. These scores are impacted by lymphedema, which is often induced by the surgical procedure and can last up to 20 years after the procedure [43], while the scores for hair loss and side effects of therapy are better than those reported in Kuwait [42] and worse than those suggested by Arndt et al. [39]. Changes in scores are often caused by chemotherapy in the first year. Nevertheless, chemotherapy's adverse effects may persist for 5 to 10 years after diagnosis [44]. Most positive changes in quality of life occur between one and two years after treatment [45]. On the other hand, socioeconomic characteristics play an inescapable role in determining the quality of life. Indeed, the notable correlations with GHS/QOL in this study were employment status, distance from home to the treatment center, medical coverage, discrimination, and income adjustment after the illness.

The univariate analysis of this study pointed to a significant association between GHS/QOL and discrimination. One in ten cancer survivors reported experiencing discrimination in at least one area of their daily life. For a third of them (36%), this discrimination was first experienced in their family circle. Half (50%) said they had experienced discrimination in their close social circle (friends, relations, etc.). Finally, a third (32%) said they had experienced discrimination in their professional environment and 46% in other social environments [46]. The ability of cancer patients to maintain or return to work may be affected by functional or psychological limitations resulting from the disease. In fact, feeling discriminated against by the employer is associated with an increase in the probability of job exit of about 11% for both men and women [46]. Admittedly, perceived personal discrimination is directly associated with a lower physical quality of life [47]. This can be explained by the myth that cancer is contagious and can always lead to death [48].

Univariate analysis of current work suggested that GHS/QOL was positively associated with occupational status, which is consistent with previous studies [49, 50]. Unemployment was observed in 36.5% of breast cancer survivors two years prior to diagnosis and 40% in controls. In contrast, during the first five years after diagnosis, the unemployment rate was significantly higher in the cancer survivor group than in controls. On the other hand, between 6 and 8 years, the difference was not significant [51], which is due to the detrimental effects of treatments, in this case, mastectomy plus axillary curage, which impair the survivor's ability to work [52]. Previous research has shown that axillary surgery is associated with employment status [53]. In contrast, Maunsell et al. attested that lymph node biopsy was not significant with unemployment six years after diagnosis [54]. Studies have indicated that work has many beneficial effects on quality of life [52]. A Canadian study illustrated that 79% of breast cancer survivors worked three years after diagnosis [55], while in a Norwegian study, 82% of nondisabled survivors continued to work up to 14 years after diagnosis [56].

Furthermore, our study's data unveiled a significant correlation between GHS/QOL and income adjustment after illness, and our results corroborate with those of other studies. A quarter of a sample of 3133 long-term breast cancer survivors reported being worse off financially because of their breast cancer. 12% reported medical debts after four years of diagnosis [57]. In other words, the financial burden of breast cancer represented on average 98%, 41%, and 26% of the monthly income of breast cancer survivors whose annual household income was < or =$30,000, $30,001–$60,000, and >$60,000, respectively [58]. Another study showed that there is a loss or reduction in average income of 21% one year after diagnosis [59]. A variety of previous studies have indicated that cancer-induced financial hardship has been associated with poor quality of life [60–64]. Rural survivors were more likely to report a loss of income compared to urban survivors [65]. Consistent with our findings, a prospective, observational, population, and health systems-based cohort study reported that 48% of the cohort had some degree of difficulty living on current household income [63]. In fact, despite the so-called global coverage by a medical cover, there are leftovers to be paid by the survivors mainly due to the expenses of consultations, transport, prescription, food supplements, and accommodation. Consistent with previous studies, this work has demonstrated that health insurance coverage is associated with breast cancer survivors' quality of life [66, 67]. Besides, Henry Y et al. confirmed that health insurance status was a significant predictor of the primary lymph node tumor's advanced stage and size [68].

The specificity of cancers and their optimal management may lead the patient, according to the referring physician's opinion, to go to a more distant treatment center because of the competence and level of expertise. An earlier study recorded that remoteness had a significant negative relationship with survivors' quality of life [69]. These results are consistent with those of the current study. Lenhard et al. suggest that late diagnosis was not associated with increased travel time to the diagnostic center [70], while another study confirmed that advanced diagnoses had longer average travel distances than early-stage diagnoses [71]. Other studies have implied that distance traveled to the treatment center was statistically correlated with survival [72, 73], and distance to the treatment center was a predictor of mortality [74]. Multivariate analysis of this work revealed that income adjustment after the illness was a significant predictor of GHS/QOL. A recent study using data from the National Health Survey indicated that increased financial burden was an independent predictor of the low quality of life for cancer survivors [64]. Another significant predictor of GHS/QOL suggested by our study was discrimination. These data are consistent with previous studies [54, 74].

Our research's main limitation was that it was a study based on a single tertiary cancer center; therefore, the results could not be generalized to the population of women with breast cancer in Morocco. Moreover, it did not include single women, which influenced the percentage of age groups. Additionally, the high incidence of illiteracy among participants did not allow for self-administered use of the questionnaire except for a minority of participants. Another limitation was the cross-sectional nature of this study that measured the HRQoL of women with breast cancer in their first two years of survival. As a result, there were no baseline and no data to compare their HRQoL before cancer interventions.

## 5. Conclusion

This study unveiled that half of breast cancer survivors, two years after diagnosis, have a good overall quality of life. The EORTC QLQ-C30 functional scales were good, and all symptom scales were slightly impaired except for the moderately impaired aspect of financial hardship.

In addition, certain socioeconomic characteristics are strongly associated with GHS/QOL, namely, discrimination and the adaptation of income to survivors' daily demands. In sum, income adjustment after illness and discrimination were significant predictors of GHS/QOL. The data suggest that a financial support program should be put in place to alleviate breast cancer survivors' financial constraints. Thus, strategies were developed to address discrimination in the community by implementing education, awareness, and antidiscrimination policies.

## Figures and Tables

**Table 1:** Sociodemographic characteristics of the study population.

Characteristics		Number	Percentage
Age	Under 25 years old	4	1.3
	26 to 45 years old	90	29.6
	46 to 65 years old	188	61.8
	Over 65 years old	22	7.2

Place of residence	Urban	212	69.7
	Rural	91	29.9

Distance from home to center	Less than 50 km	124	40.8
	Between 51 and 200 km	110	36.2
	Between 201 and 350 km	62	20.4
	Between 351 and 500 km	4	1.3
	More than 501 km	4	1.3

Level of education	Illiterate	158	52.0
	Primary	82	27.0
	Secondary	46	15.1
	Superior	17	5.6

Marital status	Bride	304	100

Professional status	Professional activity	76	25.0
	No professional activity	228	75.0

Social security	RAMED	208	68.4
	CNOPS	62	20.4
	CNSS	31	10.2
	Insurance	1	0.3

Discrimination	Yes, often	21	6.9
	Yes, a few times	75	24.7
	No, never	208	68.4

Monthly income before the illness	No income	132	43.4
	Less than 2500 DHS	115	37.5
	Between 2501 DHS and 5000 DHS	42	13.8
	Between 5001 DHS and 7500 DHS	11	3.6

Changes in monthly income after the illness	Increase	14	4.6
	Decrease	102	33.6
	Stability	188	61.8

Reason attributed to change in monthly income	No change	130	42.8
	At my state of health	148	48.7
	Has a reason independent of my state of health	26	8.6

Adjustment of monthly income after illness	With a lot of difficulty	116	38.2
	With difficulty	131	43.1
	With conveniently	57	18.8

RAMED: Insurance for low-income patients; CNOPS: National Fund for Social Security Organizations; CNSS: National Social Security Fund.

**Table 2:** EORTC QLQ-C30 scores and perceived level of quality of life by breast cancer survivors.

EORTC QLQ-C30 variables	No of items	Mean	Standard deviation
Global health status/QoL	2	57.2	25.4
Functional scales			
PF	5	72.6	28.0
RF	2	71.6	31.2
EF	4	51.2	31.3
CF	2	78.4	30.5
SF	2	84.5	29.3
Symptom scales/items			
FA	3	33.3	30.1
NV	2	11.7	26.5
PA	2	34.3	32.6
DY	1	32.0	38.7
SL	1	27.9	37.1
AP	1	19.3	32.6
CO	1	12.9	28.8
DI	1	12.1	25.6
FI	1	54.1	39.9

PF: physical functioning; RF: role functioning; EF: emotional functioning; CF: cognitive functioning; SF: social functioning; FA: fatigue; NV: nausea and vomiting; PA: pain; DY: dyspnea; SL: insomnia; AP: appetite loss; CO: constipation; DI: diarrhea; FI: financial difficulties.

**Table 3:** EORTC QLQ-BR 23 scores and perceived quality of life level of breast cancer survivors.

Variables	No of items	Mean	Standard deviation
Functional scales			
BRBI	4	58.7	31.7
BRSEF	2	49.7	29.7
BRSEE	1	52.0	29.1
BRFU	1	52.2	42.4

Symptom scales/items			
BRST	7	27.9	27.7
BRBS	4	35.7	28.2
BRAS	3	45.2	33.4
BRHL	1	22.2	36.8

BRBI: body image; BRSEF: sexual functioning; BRSEE: sexual enjoyment; BRFU: future perspective; BRST: systemic therapy side effects; BRBS: breast symptoms; BRAS: arm symptoms; BRHL: upset by hair loss.

**Table 4:** Results of multiple linear regression between global health status/QOL and socioeconomic in breast cancer survivors.

Independent variable	Global health status univariate analysis multivariate analysis				
	*β*	*P* value	*β*	*P* value	(95.0% CI)
Age	0.062	0.278	—	—	—
Place of residence	−0.016	0.785	—	—	—
Level of education	0.006	0.911	—	—	—
Professional status	0.114	0.047	0.119	0.051	(−0.016 to 13.798)
Distance from home to center	−0.140	0.015	−0.082	0.131	(−5.550 to 0.721)
Social security	0.113	0.050	0.062	0.283	(−1.903 to 6.485)
Discrimination	0.268	0.001	0.216	0.001	(4.552 to 13.351)
Monthly income before the illness	0.014	0.815	—	—	—
Changes in monthly income after the illness	−0.093	0.106	−0.019	0.738	(−6.107 to 4.333)
Reason attributed to change in monthly income	−0.021	0.712	—	—	—
Adjustment of monthly income after illness	0.329	0.001	0.259	0.001	(2.744 to 7.124)

## Data Availability

All data generated or analysed during this study are included within this article.

## Abbreviations

NOI: National Institute of Oncology
EORTC: European Research Organization for Cancer Treatment
GHS/QOL: Global health status/quality of life
RAMED: Insurance for low-income patients
CNOPS: National Fund for Social Security Organizations
CNSS: National Social Security Fund.
